# Cutaneous ureterostomy following robot-assisted radical cystectomy: a multicenter comparative study of transperitoneal versus retroperitoneal techniques

**DOI:** 10.1007/s00345-024-05300-x

**Published:** 2024-10-23

**Authors:** Yutaro Sasaki, Yasuyo Yamamoto, Kyotaro Fukuta, Kazuyoshi Izumi, Fumiya Kadoriku, Kei Daizumoto, Keito Shiozaki, Ryotaro Tomida, Yoshito Kusuhara, Tomoya Fukawa, Yutaka Yanagihara, Kunihisa Yamaguchi, Hirofumi Izaki, Masayuki Takahashi, Kenjiro Okamoto, Masahito Yamanaka, Junya Furukawa

**Affiliations:** 1 Department of Urology, Tokushima University Graduate School of Biomedical Sciences, 3-18- 15 Kuramoto-cho, Tokushima, 770-8503 Japan; 2 Department of Urology, Tokushima Prefectural Central Hospital, 1-10-3 Kuramoto-cho, Tokushima, 770-8539 Japan; 3 Department of Urology, Takamatsu Red Cross Hospital, 4-1-3 Ban-cho, Takamatsu, 760-0017 Japan; 4 Department of Urology, Ehime Prefectural Central Hospital, 83 Kasuga-machi, Matsuyama, 790-0024 Japan

**Keywords:** Cutaneous ureterostomy, Robot-assisted radical cystectomy, Bladder cancer, Transperitoneal technique, Retroperitoneal technique

## Abstract

**Background:**

The aim of this study was to evaluate the differences in perioperative outcomes between transperitoneal and retroperitoneal techniques in cutaneous ureterostomy (CUS).

**Methods:**

Between 2018 and 2023, 55 patients underwent CUS following robot-assisted radical cystectomy. Among the 55 patients, we compared 33 patients who underwent transperitoneal CUS (t-CUS) and 22 who underwent retroperitoneal CUS (r-CUS).

**Results:**

Compared with the r-CUS group, the t-CUS group had significantly shorter operative times (*p* < 0.001); significantly less estimated blood loss (*p* < 0.001); and significantly lower incidence of complications (Clavien–Dindo classification grade ≤ 2) within 30 days (*p* = 0.005). Unexpectedly, the incidence of ileus within 30 days was lower, though the difference was not statistically significant (*p* = 0.064). During the median follow-up period of 24.3 months, no ileus was observed in either group after 30 days postoperatively. There was no significant difference in the stent-free rate between the groups (*p* = 0.449). There were also no significant differences in the rates of change in estimated glomerular filtration rate from preoperatively at 3, 6, 12, and 24 months postoperatively between the groups (*p* = 0.590, *p* = 0.627, *p* = 0.741, and *p* = 0.778, respectively).

**Conclusions:**

Compared with r-CUS, t-CUS was associated with a shorter operative time and lower incidence of perioperative complications, including gastrointestinal complications. We believe that t-CUS can be performed safely and effectively.

**Supplementary Information:**

The online version contains supplementary material available at 10.1007/s00345-024-05300-x.

## Introduction

As the patient population that requires radical cystectomy becomes increasingly older and frail, there has been renewed interest in cutaneous ureterostomy (CUS), which has a lower risk of perioperative complications compared with ileal conduit or neobladder [1]. Robotic-assisted radical cystectomy (RARC) has improved surgical and oncological safety and efficacy compared with open radical cystectomy [2–4]. However, although intracorporeal urinary diversion following RARC has a similar incidence of complications compared with CUS, intracorporeal urinary diversion has disadvantages, such as longer operative time and increased blood loss [5]. Therefore, compared with intracorporeal urinary diversion, combining RARC with CUS is a minimally invasive surgical approach in the treatment of bladder cancer that is more acceptable to older patients, frail individuals, and those with multiple comorbidities [4, 6]. In CUS, the ureter can be routed retroperitoneally or transperitoneally. Although each technique has advantages and disadvantages, no studies have directly compared these techniques, and they have been discussed rarely. Therefore, we evaluated the differences in perioperative outcomes between transperitoneal CUS (t-CUS) and retroperitoneal CUS (r-CUS).

## Materials and methods

### The study population and variables

Between April 2018 and July 2023, 364 patients underwent RARC at Tokushima University Hospital, Tokushima Prefectural Central Hospital, Takamatsu Red Closs Hospital, and Ehime Prefectural Central Hospital. Of these 364 patients, 55 underwent CUS for urinary diversion. The surgical procedures were performed by 14 console surgeons, each with experience performing over 200 robot-assisted surgeries. The indications for performing CUS were the presence of comorbidities, history of radiotherapy to the abdomen, and patients’ preferences. Whether the CUS was created transperitoneally or retroperitoneally was determined by the surgeon’s preference. Among the 55 patients, we compared 33 patients who underwent t-CUS and 22 who underwent r-CUS to investigate the impact of each technique on surgical outcomes. Data were reviewed for the patients’ characteristics (age, sex, body mass index, estimated glomerular filtration rate (eGFR), Eastern Cooperative Oncology Group performance status, American Society of Anesthesiologists physical status, clinical stage, bilateral CUS, lymph node dissection, neoadjuvant chemotherapy, and adjuvant chemotherapy), surgical outcomes (operative time, RARC time, CUS time, estimated blood loss, transfusion, pathological stage, time to liquid intake, time to solid food intake, length of hospital stay, complications, stent-free rate, follow-up duration, readmission owing to urinary tract infection (UTI), and the number of readmissions owing to UTI). RARC time was defined as the time required for radical cystectomy and lymph node dissection. Complications were categorized in accordance with the Clavien–Dindo classification system. Grade ≥ 3 complications were defined as major complications, and grade ≤ 2 complications were defined as minor complications. Changes in eGFR were compared preoperatively and 3, 6, 12, and 24 months postoperatively. Readmission rates and the number of hospitalizations owing to UTI during the follow-up period were also compared between the two groups.

### Surgical techniques

**Fig. 1:**
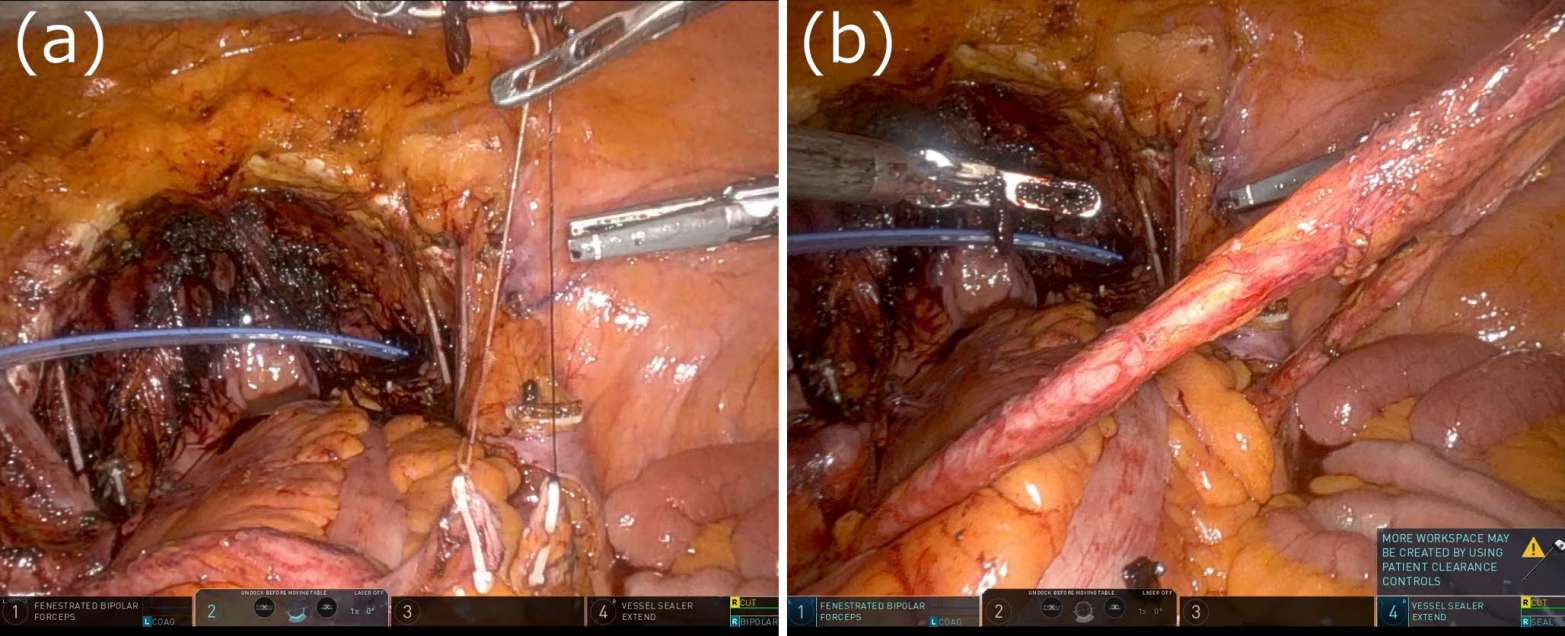
Surgical images showing transperitoneal CUS. (**a**) The patient-side surgeon inserts Pean forceps transperitoneally from the stoma site. (**b**) The patient-side surgeon retracts the ureter out of the body. CUS, cutaneous ureterostomy

**Fig. 2:**
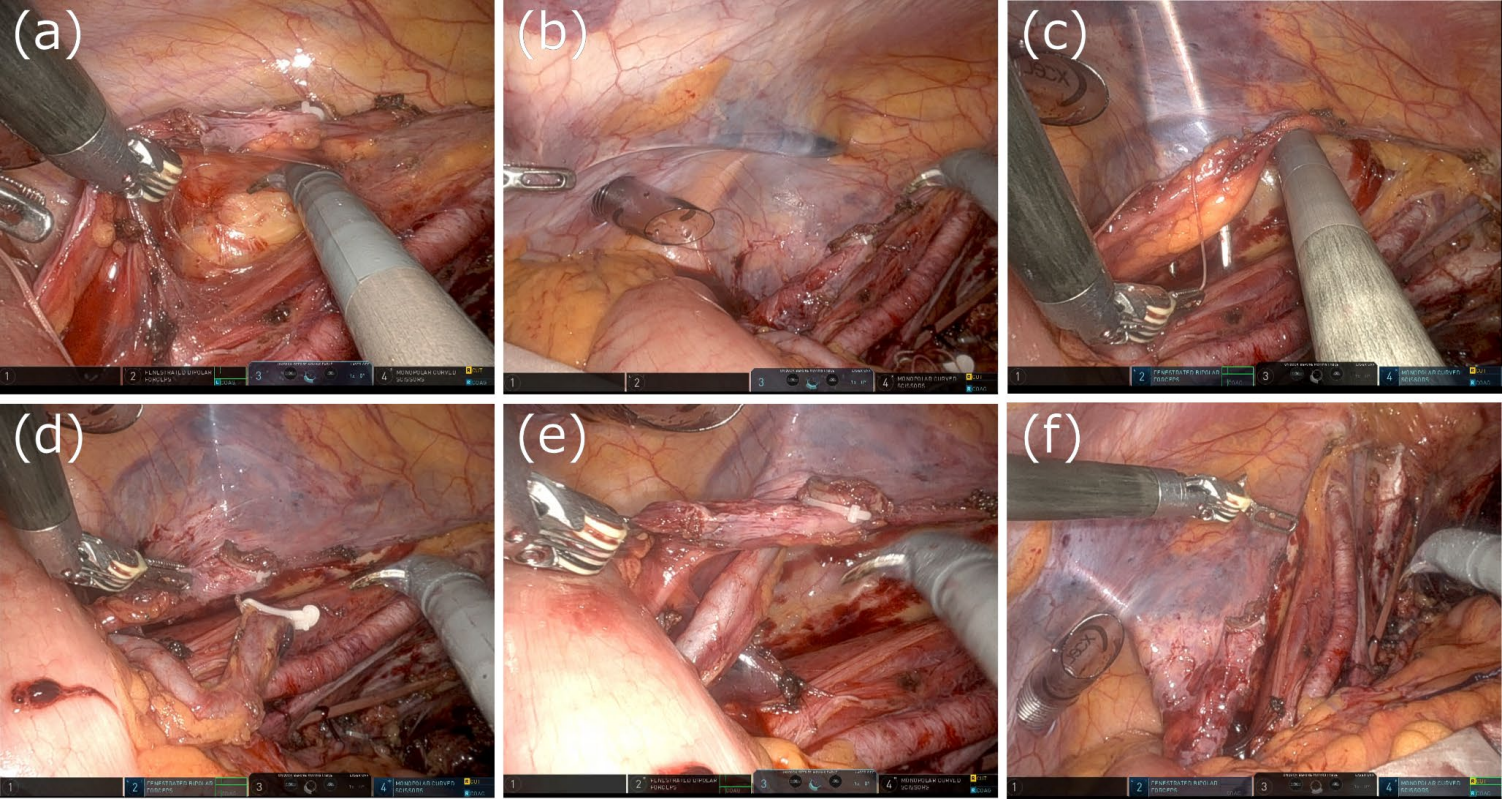
Surgical images showing retroperitoneal CUS. (**a**) The console surgeon expands the retroperitoneal space laterally toward the stoma site. (**b**) The patient-side surgeon expands the retroperitoneal space near the stoma site using a trocar. (**c**) The patient-side surgeon then inserts Pean forceps retroperitoneally from the stoma site. (**d**) The patient-side surgeon retracts the ureter out of the body. (**e**) The console surgeon checks to make sure the left ureter is not bent. (**f**) Retroperitonealization of the ureter is completed. CUS, cutaneous ureterostomy

The surgical procedure for CUS is described below. Following radical cystectomy and lymph node dissection via a transperitoneal approach, the console surgeon first dissects the ureter toward the kidney to prevent bending or stretching of the ureter. In the case of t-CUS, the patient-side surgeon makes an incision at the stoma site, which is marked before surgery, inserts Pean forceps transperitoneally, grasps the ureter, and retracts it out of the body (Fig. 1). In the case of r-CUS, the patient-side surgeon makes an incision at the stoma site as for t-CUS. The console surgeon then expands the retroperitoneal space laterally toward the stoma site. In ipsilateral CUS, the left ureter is tunneled behind the sigmoid colon. Because it is difficult for the console surgeon to expand the retroperitoneal space near the stoma site, the patient-side surgeon expands the retroperitoneal space using Pean forceps or a trocar. Then, the patient-side surgeon inserts Pean forceps retroperitoneally from the stoma site and retracts the ureter out of the body (Fig. 2).

The stoma was created using the Toyoda method in both groups [7]. Patients in both groups received standard postoperative care, not enhanced recovery after surgery protocols. The single J stent was removed 2–4 weeks after surgery. The stent was reinserted in the event of UTI or decreased renal function. In such cases, patients underwent stent replacement every 4 weeks.

### Statistical analysis

All quantitative variables are expressed as median [interquartile range]. Quantitative variables were evaluated using the Mann–Whitney U test, and categorical variables were analyzed using Pearson’s chi-square test. A p-value of < 0.05 was considered statistically significant. All statistical analyses were performed with EZR (Saitama Medical Center, Jichi Medical University, Saitama, Japan), which is a graphical user interface for R (ver.4.1.2; www.r-project.org).

## Results

Table 1 shows the patients’ characteristics between the t-CUS and r-CUS groups. Age, sex, body mass index, and preoperative eGFR were similar between the groups. In the t-CUS group, there were 16 patients of bilateral CUS and 17 patients of ipsilateral CUS, and in the r-CUS group, there were eight patients of bilateral CUS and 14 patients of ipsilateral CUS (*p* = 0.542).

**Table 1:** Comparison of patient characteristics between transperitoneal CUS and retroperitoneal CUS groups

	t-CUS		*r*-CUS		*p*-value
	(*n*=33)		(*n*=22)		
Age, years, median (IQR)	78 (71–84)		75 (71–82)		0.434
Male, n (%)	18	(55)	17	(77)	0.153
BMI, kg/m^2^, median (IQR)	22.1 (19.8–25.0)		23.5 (22.3–25.6)		0.131
eGFR, mL/min/1.73m^2^, median (IQR)	55.8 (39.1-77.2)		43.7 (33.5-62.3)		0.131
ECOG-PS ≥2, n (%)	7	(21)	2	(9)	0.413
ASA-PS ≥3, n (%)	11	(33)	12	(55)	0.199
Clinical tumor stage, n (%)					
cTis/T1	11	(33)	4	(18)	0.087
cT2	11	(33)	14	(64)	
cT3/T4	11	(33)	4	(18)	
Clinical nodal stage ≥1, n (%)	8	(24)	1	(5)	0.118
Bilateral CUS, n (%)	16	(48)	8	(36)	0.542
Lymph node dissection, n (%)	23	(70)	19	(86)	0.271
Neoadjuvant chemotherapy, n (%)	6	(18)	9	(41)	0.122
Adjuvant chemotherapy, n (%)	4	(12)	3	(14)	1.000

CUS: cutaneous ureterostomy, t-CUS: transperitoneal CUS, r-CUS: retroperitoneal CUS, IQR: interquartile range, BMI: body mass index, eGFR: estimated glomerular filtration rate, ECOG-PS: Eastern Cooperative Oncology Group performance status, ASA-PS: American Society of Anesthesiologists physical status classification

Table 2 shows the surgical outcomes between the groups. The operative time was significantly shorter in the t-CUS group than that in the r-CUS group (259 [233–326] vs. 370 [332–470] min, respectively; *p* < 0.001), and the RARC time was also shorter in the t-CUS group (193 [158–224] vs. 227 [201–311] min, respectively; *p* = 0.015). The findings were similar for CUS time, which was significantly shorter in the t-CUS group vs. the r-CUS group (64 [51–83] vs. 99 [60–147] min, respectively; *p* = 0.012). Although estimated blood loss was significantly lower in the t-CUS group than that in the r-CUS group (104 [50–200] vs. 288 [153–490] ml, respectively; *p* < 0.001), transfusion rates were similar between the groups (15% vs. 36%, respectively; *p* = 0.136).

**Table 2:** Comparison of surgical outcomes between transperitoneal CUS and retroperitoneal CUS groups

	t-CUS		*r*-CUS		*p*-value
	(*n*=33)		(*n*=22)		
Operative time, min, median (IQR)	259 (233–326)		370 (332–470)		<0.001
RARC time, min, median (IQR)	193 (158–224)		227 (201–311)		0.015
CUS time, min, median (IQR)	64 (51–83)		99 (60–147)		0.012
Estimated blood loss, ml, median (IQR)	104 (50–200)		288 (153–490)		<0.001
Transfusion, n (%)	5	(15)	8	(36)	0.136
Pathological tumor stage, n (%)					
pT0	1	(3)	5	(23)	0.137
pTa/Tis/T1	9	(27)	6	(27)	
pT2	6	(18)	3	(14)	
pT3/T4	17	(52)	8	(36)	
Positive lymph node, n (%)	7	(21)	6	(27)	0.846
Time to liquid intake, POD, median (IQR)	1 (1–1)		1 (1–1)		0.701
Time to solid food intake, POD, median (IQR)	2 (2–2)		2 (2–3)		0.180
Hospital stay, days, median (IQR)	20 (16–30)		27 (23–33)		0.031
30-day complications					
Minor complications, n (%)	5	(15)	12	(55)	0.005
Major complications, n (%)	6	(18)	3	(14)	0.941
90-day complications					
Minor complications, n (%)	6	(18)	3	(14)	0.941
Major complications, n (%)	2	(6)	1	(5)	1.000
Stent-free rate, n (%)	5	(15)	6	(27)	0.449
Follow-up, months, median (IQR)	22.0 (8.3–42.2)		27.6 (11.6–43.5)		0.345
Readmission owing to UTI, n (%)	15	(45)	11	(50)	0.956
Number of readmissions owing to UTI	1.0 (1.0–2.0)		2.5 (2.0–3.0)		0.010

CUS: cutaneous ureterostomy, t-CUS: transperitoneal CUS, r-CUS: retroperitoneal CUS, IQR: interquartile range, RARC: robot-assisted radical cystectomy, POD: postoperative day, UTI: urinary tract infection, min, minutes

There was no significant difference between the t-CUS and r-CUS groups in the incidence of major complications within 30 days (18% vs. 14%, respectively; *p* = 0.941). However, the incidence of minor complication within 30 days was significantly higher in the r-CUS vs. t-CUS groups (15% vs. 55%, respectively; *p* = 0.005). Supplemental Table 1 shows detailed information on the perioperative complications between the groups. Ileus within 30 days occurred in one patient in the t-CUS group (grade 2) and five in the r-CUS group (four grade 2, one grade 3a). In other words, the incidence of all-grade ileus within 30 days was higher in the r-CUS group than that in the t-CUS group, although the difference was not statistically significant (3% vs. 23%, respectively; *p* = 0.064). There was no significant difference in the stent-free rate between the t-CUS and r-CUS groups (15% vs. 27%, respectively; *p* = 0.449). The median follow-up period also did not differ between the t-CUS and r-CUS groups (22 [8–42] vs. 28 [12–44] months, respectively; *p* = 0.345). During follow-up, readmission rates owing to UTI were similar between the t-CUS and r-CUS groups (45% vs. 50%, respectively; *p* = 0.956). However, the number of readmissions owing to UTI was significantly higher in the r-CUS vs. t-CUS groups (1.0 [1.0–2.0] vs. 2.5 [2.0–3.0], respectively; *p* = 0.010).

**Fig. 3:**
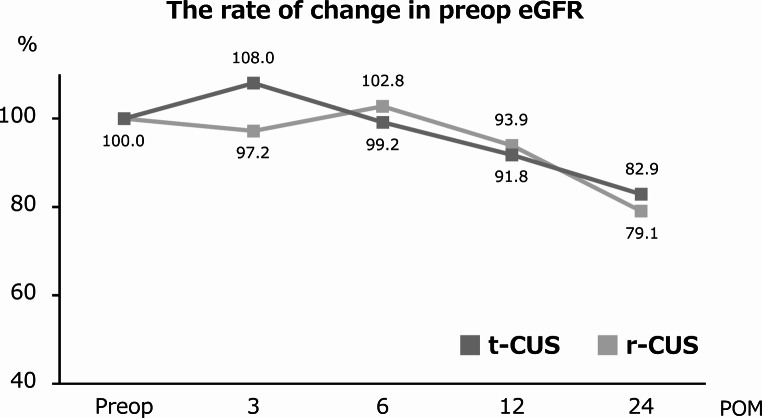
The rate of change in eGFR before and after surgery between the transperitoneal CUS and retroperitoneal CUS groups. CUS, cutaneous ureterostomy; t- transperitoneal; r-, retroperitoneal; eGFR, estimated glomerular filtration rate; Preop, preoperatively; POM, postoperative months

Figure 3 shows the preoperative and postoperative changes in eGFR. There were no significant differences in the rates of change in eGFR from preoperatively at 3, 6, 12, and 24 months postoperatively between the t-CUS and r-CUS groups (*p* = 0.590, *p* = 0.627, *p* = 0.741, and *p* = 0.778, respectively). Supplemental Fig. 1 shows the Kaplan–Meier curves for the t-CUS and r-CUS groups. The 3-year survival rates did not differ between the t-CUS group and r-CUS group, respectively: overall survival: 55% vs. 59%, *p* = 0.663; cancer-specific survival: 69% vs. 75%, *p* = 0.351; and recurrence-free survival: 62% vs. 55%, *p* = 0.993.

## Discussion

The advantages of CUS compared with urinary diversions, such as ileal conduit or neobladder, are less invasiveness, fewer gastrointestinal complications, shorter operative time, less blood loss, and shorter hospital stay [1, 5, 8]. In contrast, the disadvantage of CUS is the low stent-free rate, which is associated with an increased incidence of UTI and may impair patients’ quality of life [8]. In CUS cases that do not become stent-free, the burden on patients and their families forced to undergo regular stent replacement cannot be underestimated [5]. This is especially true for frail and dependent older patients, or those who have difficulty making regular trips to a hospital [1]. However, this issue is rarely addressed in patients’ health-related quality of life questionnaires [1, 9].

The gold standard for urinary diversion following radical cystectomy is the ileal conduit. We use intracorporeal ileal conduits in many patients because of the feasibility of the procedure and the tolerability of perioperative complications [5]. Nonetheless, CUS, an alternative form of urinary diversion, is indicated in some patients (e.g., history of pelvic radiation therapy, inflammatory bowel disease, multiple comorbidities, and older or frail patients) [4, 6, 10]. In these patients, shorter operative times and fewer complications are desirable. Preserving renal function after surgery is also important, given the possibility of adjuvant chemotherapy.

In CUS, the ureter can be routed retroperitoneally or transperitoneally. The advantages of t-CUS, in which the ureter is not retroperitonealized, include short operative time and feasibility even if the ureter is short. The disadvantage of t-CUS is that the ureter may become entangled with the intestine, causing ureteral or intestinal obstruction [11, 12]. The advantages of r-CUS, in which the ureter is retroperitonealized, include the possibility of fewer gastrointestinal complications compared with t-CUS and the ease of performing total nephroureterectomy for ureteral cancer [13]. The disadvantages of r-CUS are the long operative time and the difficulty routing the ureter retroperitoneally when the ureteral length is insufficient [13]. In this case, an overly stretched ureter may cause insufficient blood flow, leading to ureteral stenosis.

In the present study, the operative time and RARC time were significantly shorter in the t-CUS group compared with the r-CUS group. The estimated blood loss was also significantly less in the t-CUS vs. r-CUS groups. The shorter RARC time may have resulted from a slightly lower rate of lymph node dissection in the t-CUS vs. r-CUS groups. Additionally, compared with the t-CUS group, the r-CUS group had a median CUS time of 35 min longer. This reflects the time required for retroperitonealization of the ureter. The incidence of minor complications within 30 days was significantly higher in the r-CUS vs. t-CUS groups. Notably, the incidence of ileus within 30 days was higher in the r-CUS group vs. the t-CUS group, though the difference was not statistically significant (3% vs. 23%, respectively; *p* = 0.064). During the median follow-up period of 24.3 months, no ileus was observed in either group after 30 days postoperatively. Ben-David et al. evaluated 69 patients who underwent t-CUS following RARC [14]. The median age was 77 years, and 59% of the patients had an American Society of Anesthesiologists physical status score ≥ 3. The median operative time was 241 min, and the median estimated blood loss was 100 mL, which were comparable to the perioperative outcomes in our t-CUS group. In Ben-David et al.’s study, the incidence of complications within 30 days was 55%, and 10% were gastrointestinal complications (details unknown). The incidence of complications within 30–90 days was 23%, with no gastrointestinal complications.

In radical cystectomy, prolonged operative time increases both the risk of postoperative complications and the readmission rate [15, 16]. Hanna et al. reported that the operative time threshold at which the likelihood of postoperative complications increases significantly was 369 min [16]. In the present study, the median operative time in the r-CUS group was 370 min, exceeding 369 min. On the basis of these findings, we believe that the higher incidence of complications with r-CUS compared with t-CUS was mainly because of the prolonged operative time. Notably, although the rate of hospitalization owing to UTI during the observation period was similar in both groups, the median number of hospitalizations was significantly higher in the r-CUS vs. t-CUS groups (1.0 vs. 2.5, respectively; *p* = 0.010). The non-inferiority of t-CUS was also demonstrated in terms of the incidence of postoperative UTI. It is unclear why the median number of hospitalizations was higher in the r-CUS group vs. the t-CUS group. In the present study, postoperative eGFR was significantly higher in the t-CUS group compared with the r-CUS group. We believe that this is because the t-CUS group has a higher preoperative eGFR and a lower stent-free rate compared with the r-CUS group, although the difference was not statistically significant.

There are few reports on t-CUS. Furubayashi et al. reported a surgical technique for t-CUS using the transverse mesocolon [13]. In the study, nine patients underwent this procedure, and no intraoperative complications were observed. One patient developed ileus, which resolved with conservative treatment [13]. However, the report did not provide information on the stent-free rate or a comparison of surgical outcomes between t-CUS and r-CUS. In a literature search, we identified no previous reports comparing the surgical outcomes of t-CUS and r-CUS. To our knowledge, ours is the first study to compare the surgical outcomes of t-CUS and r-CUS.

The results of the present study are encouraging for urologists who perform t-CUS and are also useful for urologists who usually perform r-CUS. When the available ureteral length is insufficient, r-CUS is difficult to perform. Specifically, there are cases in which ureteral frozen section analysis was performed multiple times and cases where it was difficult to dissect the lower ureter owing to adhesions. When r-CUS is performed in such cases, we believe that an overly stretched ureter may cause insufficient blood flow, leading to ureteral stenosis. These patients can be converted to t-CUS when necessary if the ureteral length is insufficient.

The present study has limitations that must be considered. First, this was a retrospective study that involved multiple centers, and the study had a relatively small sample size. These issues may limit the generalizability of the findings. Second, CUS patients accounted for only 15.1% of all RARC patients, making it difficult to collect data from a large number of patients. Third, the quality of CUS can vary between surgeons, which may have affected the results of our study.

## Conclusion

Compared with r-CUS, t-CUS was associated with a shorter operative time and lower incidence of perioperative complications, including gastrointestinal complications. We believe that t-CUS can be performed safely and effectively.

## Electronic supplementary material

Below is the link to the electronic supplementary material.

Supplementary Material 1

Supplementary Material 2

## Data Availability

No datasets were generated or analysed during the current study.
