# Supplementary material for: Cutaneous ureterostomy following robot-assisted radical cystectomy: a multicenter comparative study of transperitoneal versus retroperitoneal techniques
Source: World J Urol. 2024 Oct 23;42(1):591. doi: 10.1007/s00345-024-05300-x (PMC11499339; doi:10.1007/s00345-024-05300-x)
Supplement: Supplementary file 2 — Supplementary Material 2 [file 345_2024_5300_MOESM2_ESM.docx]

**Supplemental Fig. 1** Kaplan–Meier curves for the transperitoneal CUS and retroperitoneal CUS groups The black line represents 33 patients who underwent transperitoneal CUS, and the pink line represents 22 patients who underwent retroperitoneal CUS. (a) OS (log-rank, p = 0.663), (b) CSS (log-rank, p = 0.351), and (c) RFS (log-rank, p = 0.993). CUS, cutaneous ureterostomy; t- transperitoneal; r-, retroperitoneal; OS, overall survival; CSS, cancer-specific survival; RFS, recurrence-free survival
